# A simplified system for the effective expression and delivery of functional mature microRNAs in mammalian cells

**DOI:** 10.1038/s41417-019-0113-y

**Published:** 2019-06-20

**Authors:** Jiaming Fan, Yixiao Feng, Ruyi Zhang, Wenwen Zhang, Yi Shu, Zongyue Zeng, Shifeng Huang, Linghuan Zhang, Bo Huang, Di Wu, Bo Zhang, Xi Wang, Yan Lei, Zhenyu Ye, Ling Zhao, Daigui Cao, Lijuan Yang, Xian Chen, Bin Liu, William Wagstaff, Fang He, Xiaoxing Wu, Jing Zhang, Jennifer Moriatis Wolf, Michael J. Lee, Rex C. Haydon, Hue H. Luu, Ailong Huang, Tong-Chuan He, Shujuan Yan

**Affiliations:** 1 Ministry of Education Key Laboratory of Diagnostic Medicine, and the School of Laboratory Medicine, Chongqing Medical University, 400016 Chongqing, China; 2 Molecular Oncology Laboratory, Department of Orthopaedic Surgery and Rehabilitation Medicine, The University of Chicago Medical Center, Chicago, IL 60637 USA; 3 The Affiliated Hospitals of Chongqing Medical University, 400016 Chongqing, China; 4 Department of Clinical Laboratory Medicine, The First Affiliated Hospital of Guiyang College of Traditional Chinese Medicine, 550001 Guiyang, China; 5 Department of Clinical Laboratory Medicine, The Second Affiliated Hospital of Nanchang University, 330006 Nanchang, China; 6 Key Laboratory of Orthopaedic Surgery of Gansu Province, and the Departments of Orthopaedic Surgery and Obstetrics and Gynecology, The First and Second Hospitals of Lanzhou University, 730030 Lanzhou, China; 7 Department of General Surgery, The Second Affiliated Hospital of Soochow University, 215004 Suzhou, China; 8 Department of Orthopaedic Surgery, Chongqing General Hospital, 400021 Chongqing, China; 9 Department of Clinical Laboratory Medicine, The Affiliated Hospital of Qingdao University, 266061 Qingdao, China; 10 School of Life Sciences, Southwest University, 400715 Chongqing, China; 11 Department of Clinical Laboratory Medicine, Guizhou Provincial People’s Hospital and Guizhou University, 550004 Guiyang, China

**Keywords:** Cancer, Biological techniques

## Abstract

MicroRNAs (miRNAs) are ~22 nucleotide noncoding RNAs that are involved in virtually all aspects of cellular process as their deregulations are associated with many pathological conditions. Mature miRNAs (mMIRs) are generated through a series of tightly-regulated nuclear and cytoplasmic processing events of the transcribed primary, precursor and mMIRs. Effective manipulations of miRNA expression enable us to gain insights into miRNA functions and to explore potential therapeutic applications. Currently, overexpression of miRNAs is achieved by using chemically-synthesized miRNA mimics, or shRNA-like stem-loop vectors to express primary or precursor miRNAs, which are limited by low transfection efficacy or rate-limiting miRNA processing. To overcome rate-limiting miRNA processing, we developed a novel strategy to express mMIRs which are driven by converging U6/H1 dual promoters. As a proof-of-concept study, we constructed mMIR expression vectors for hsa-miR-223 and hsa-Let-7a-1, and demonstrated that the expressed mMIRs effectively silenced target gene expression, specifically suppressed miRNA reporter activity, and significantly affected cell proliferation, similar to respective primary and precursor miRNAs. Furthermore, these mMIR expression vectors can be easily converted into retroviral and adenoviral vectors. Collectively, our simplified mMIR expression system should be a valuable tool to study miRNA functions and/or to deliver miRNA-based therapeutics.

## Introduction

The completion of the Human Genome Project and the advent of high-throughput deep sequencing technologies have revealed that, while eukaryotic genomes are pervasively transcribed, <2% of the human genome is transcribed into protein-coding mRNA, which leaves most of the transcribed human genome noncoding RNAs (ncRNAs) [1–6]. Based on their sizes, ncRNAs are divided into two groups: small ncRNAs (<200 nt) and long ncRNAs or lncRNAs (>200 nt) [7, 8]. While the biological functions of ncRNAs remain to be fully understood, increasing evidence suggests that ncRNAs play critical regulatory roles in numerous cellular processes [5, 7–11]. Nonetheless, small ncRNAs, such as microRNAs and small interfering RNAs (siRNAs), have received much attention since their discoveries.

MicroRNAs (miRNAs or miRs) are small noncoding RNAs of ~22 nucleotides (nt) in length, which induce gene silencing by guiding Argonaut (AGO) proteins to completely or partially complementary binding sites at the 3′ untranslated region (UTR) of target mRNAs [12–18]. The first miRNA was discovered in 1993 in *Caenorhabditis elegans* as a short RNA produced by the *lin-4* gene, which post-transcriptionally represses the *lin-14* mRNA [19–21]. Such small regulatory RNAs were later found abundantly presented in diverse animal phyla and were subsequently named microRNAs [13]. Currently, the miRNA repository miRBase lists 1917 precursor miRNAs (pMIRs) and 2654 mature miRNAs (mMIRs) for humans [22], and it has been estimated that >60% of human protein-coding genes harbor predicted miRNA target sites [23].

The short single-stranded miRNAs are initially transcribed as longer primary transcripts (or termed pri-miRNAs), containing a 60–120 nt RNA hairpin in which one of the two strands includes the mMIR[13]. The hairpin-containing pri-miRNAs are successively cleaved by two RNase III enzymes, Drosha in the nucleus and Dicer in the cytoplasm, to yield ~70 nt pMIRs and 22 nt mMIRs, respectively [13]. The pMIRs are transported to the cytoplasm via Exportin-5 and further processed by Dicer to produce a short, partially double-stranded RNA, in which one strand is the mMIR. mMIRs modulate gene expression posttranscriptionally by imperfectly binding target mRNAs in association with the AGO-containing multi-protein RNA-induced silencing complex [13]. AGOs are a large family of proteins that use single-stranded small nucleic acids as guides to complementary sequences in RNA or DNA targeted for silencing [13, 24]. The miRNA-loaded AGO forms the targeting module of the miRNA-induced silencing complex, leading to translation repression and/or degradation of targeted mRNAs [13, 25]. Nonetheless, recent evidence has revealed that miRNA processing steps may follow canonical processing routes, and/or many noncanonical miRNA biogenesis pathways, which crosstalk with other cellular pathways [17].

It is well established that miRNAs are involved in virtually every cellular process and are essential for development, cell differentiation, and homeostasis [13]. In fact, deregulation of miRNA function has been associated with human diseases [12, 26], particularly in cancers [13, 27, 28], as miRNAs can function as both oncogenes (or oncomiRs) [29] and tumor suppressors [30], although miRNA expression is generally downregulated in most cancers [13, 27, 28, 31]. Thus, it is highly desirable to effectively manipulate the exogenous miRNA expression in order to gain insights into their biological functions, and in some cases, to explore their potential therapeutic applications. Downregulation or inhibition of miRNA functions can be usually accomplished by the use of anti-miRs, antagomiRs, AMOs (anti-miRNA antisense oligonucleotides), miRNA sponges, miRNA decoys, or circularized anti-miRs, most of which are usually based on antisense molecules to bind and sequester miRNAs from their natural targets [18, 32–34]. On the other hand, upregulation or overexpression of miRNAs can be usually accomplished by the use of chemically synthesized miRNA mimics, or shRNA-like or intronic miRNA expression vectors to express the primary miRNAs (priMIRs) or pMIRs [35–37]. However, the efficacy of miRNA mimics is transient in nature and limited by transfection efficiency. The commonly used intronic miRNA expression strategy will rely on the endogenous miRNA processing efficiency and may cause cytotoxicity due to oversaturation of the RNAi machinery [37, 38]. Thus, there is an unmet need to develop fully optimized miRNA-expressing vectors for the efficient expression of miRNAs in cultured cells and animals.

In order to overcome the rate-limiting siRNA/miRNA processing machinery, here we developed a novel and simplified strategy to express mMIRs by exploiting the converging U6/H1 dual promoter-driven expression of miRNAs. We successfully used the converging U6/H1 dual promoter-driven system to express siRNAs [39, 40]. However, the asymmetric nature or imperfect complementarity of the 5p-miR and 3p-miR sequences of a given miRNA requires a different design. We overcame this challenge by inserting the transcription stop signals (a string of TTTTTAAAAA) between the 5p-miR (in sense direction) and 3p-miR (in antisense direction) sequences to terminate the transcription of 5p-miR and 3p-miR, respectively. As positive controls, we also constructed the U6-driven expression of pMIRs and the conventional priMIR expression systems.

As a proof-of-concept study, we constructed the pMIR and mMIR expression vectors for the commonly-studied hsa-miR-223 and hsa-let-7a-1. We demonstrated that the mMIRs effectively inhibited target gene expression, specifically suppressed target gene 3′-UTR-derived reporter activity, and effectively affected cell proliferation in a fashion similar to that of the respective priMIR and pMIRs expression systems in human cell lines. Furthermore, our mMIR expression vector was constructed on the bases of retroviral transfer and adenoviral shuttle vectors. Thus, the final mMIR expression constructs can be easily converted into recombinant retrovirus for stable expression or recombinant adenovirus for effective transient expression in vitro and in vivo. Collectively, our results demonstrate that the simplified mMIR expression system is user-friendly and reproducibly effective, which should be a valuable resource for studying miRNA functions and/or exploring potential applications of miRNA-based therapeutics.

## Materials and methods

### Cell culture and chemicals

HEK-293 and human colon cancer cell line HCT116 were obtained from the American Type Culture Collection (Manassas, VA, USA). The cells were maintained in the DMEM contained 10% fetal bovine serum, 100 μg streptomycin and 100 units of penicillin at 37 °C in 5% CO_2_ as previously reported [41–44]. Unless indicated otherwise, all chemicals were purchased from Sigma-Aldrich (St. Louis, MO, USA) or Thermo Fisher Scientific (Waltham, MA, USA).

### Construction of the three types of expression vectors for hsa-miR-223 (MIR223) and hsa-let-7a-1 (MIRLET7A1)

**Fig. 1:**
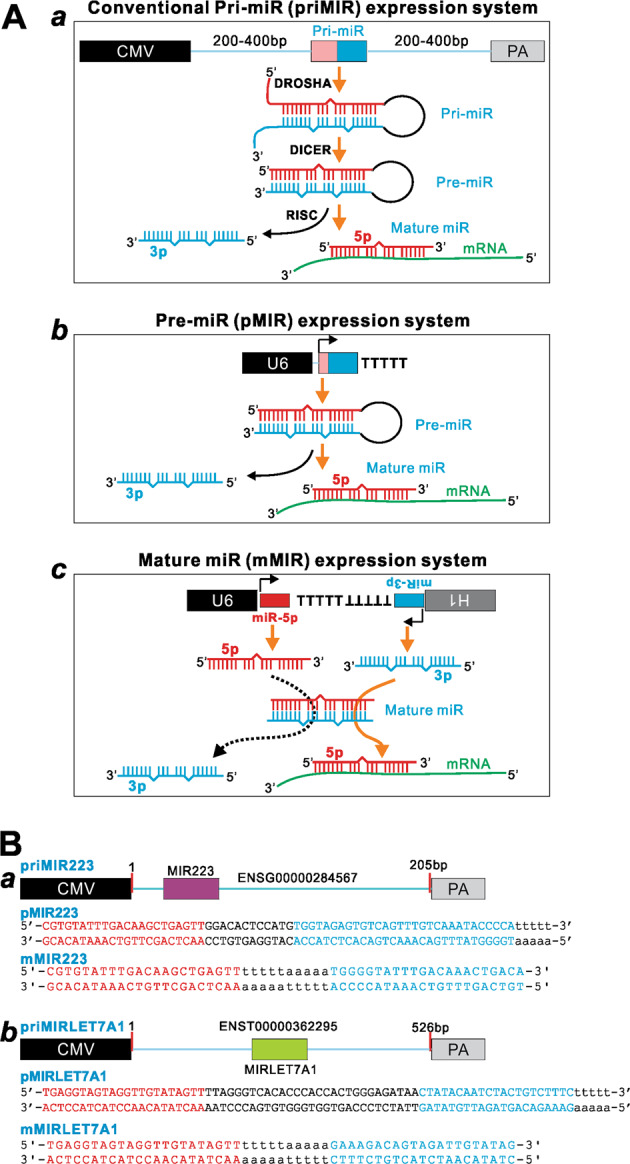
Schematic representation of the three miRNA expression systems compared in this study. **a** Schematic representation of the three expression systems and possible modes of their processing and actions. The conventional primary miR (priMIR) expression system consists of cloning 200–500 bp genomic fragment surrounding the miR transcript unit under the CMV promoter (*a*). The precursor miR (pMIR) expression system consists of the U6-driven expression of pre-miR sequence followed by a string of UUUUU (or TTTTT in the diagram) as the transcriptional termination signal (*b*). The mature miR (mMIR) expression system is composed of the convergent U6 and H1 promoter-driven the expression of mature miR-5p and miR-3p in opposing directions, respectively, with the respective transcriptional termination signal (TTTTT or UUUUU) separating the U6 and H1 expression units. **b** Two representative miRs selected for the proof-of-principal study. The schematic genomic fragments and detailed miR sequences are shown for the tested three expression constructs of hsa-miR-223 (MIR223) (*a*) and hsa-miR-let-7a-1 (MIRLET7A1) (*b*). miR, miRNA, or microRNA; CMV, cytomegalovirus promoter; U6, human U6 promoter; H1, human H1 promoter; PA, polyadenylation signal for terminating transcription

As illustrated in Fig. 1, the conventional priMIR expression vector was constructed on our home-made adenoviral shuttle vector pAdTrace-TOX [45–47], which contains the CMV promoter-driven expression cassette and SV40 Pa. Genomic DNA fragments containing primary hsa-miR-223 (MIR223) and hsa-let-7a-1 (MIRLET7A1) were PCR amplified from HEK-293 genomic DNA and subsequently cloned into the pAdTrace-OK vector, resulting in priMIR223 and priMIRLET7A1 expression vectors, respectively.

To express the precursor miRs hsa-miR-223 (pMIR223) and hsa-let-7a-1 (pMIRLET7A1), we first engineered the retroviral pSEB-pmiR and adenoviral shuttle pAdTrace-pmiR vectors on the base of our previously characterized pSOS system [39], through the Gibson Assembly system (New England Biolabs, or NEB, Ipswich, MA) as reported [40] (Supplemental Fig. 1a, b). These vectors contain a U6 promoter followed by Mlu*I* and Hind*III* cloning sites. Subsequently, we cloned oligo cassettes containing the pmiR sequences for hsa-miR-223 and hsa-let-7a-1, followed by the TTTTT transcription termination signal, into the Mlu*I* and Hind*III* cloning sites, resulting in pMIR223 and pMIRLET7A1.

For expressing mMIR’s miR-5p and miR-3p simultaneously in one vector, we engineered the retroviral pSEB-miR and adenoviral shuttle pAdTrace-miR vectors on the base of the pSOS system [39] through the Gibson Assembly system (NEB) [40] (Supplemental Fig. 2a, b). In these vectors, the expression of mature miR-5p and miR-3p is driven by the convergent U6 and H1 promoters in opposing directions, respectively, with respective transcriptional termination signals (TTTTT or UUUUU) inserted between the mature miRs. Accordingly, oligo cassettes containing the mature miR-5p and miR-3p sequences of hsa-miR-223 (mMIR223) and hsa-let-7a-1 (mMIRLET7A1) were cloned into the Sal*I* and Hind*III* sites of the pAdTrace-miR and pSEB-miR vectors, resulting in mMIR223 and mMIRLET7A1 constructs, respectively. All cloning junctions and oligo cloning-derived vectors were verified by DNA sequencing. Detailed information about the reported constructs and/or the reported vectors is available upon request.

### Cell transfection

For the reported studies, freshly seeded subconfluent cells were transfected by using the linear polyethylenimine (PEI)-based Transporter 5™ Transfection Reagent (Polysciences, Inc., Warrington, PA) according to the manufacturer’s instructions. At the indicated time points, the transfected cells were collected for various assays described below.

### Total RNA isolation and touchdown quantitative real-time PCR (TqPCR)

At the endpoint, the transfected cells were subjected to total RNA isolation by using NucleoZOL Reagent (Takara Bio USA, Mountain View, CA) according to the manufacturer’s introduction. For qPCR analysis of mRNA transcripts, total RNA was used for reverse transcription with the hexamer and M-MuLV (NEB). The cDNA products were diluted as templates for qPCR. The qPCR primers were designed by Primer3 Plus program [48]. For assessing the miR expression levels mediated by the three expression systems, the reverse transcription reactions were carried out by using miR-specific reverse primers that were complementary with the 3′-end six nucleotides of mature miR-5p and/or miR-3p, preceded with a 44-nt artificial stem-loop sequence. SYBR green-based quantitative real-time PCR analysis was performed by following our previously optimized TqPCR protocol [49]. The qPCR reactions were done in triplicate. All expression values were normalized to the reference gene *GAPDH* expression by using the 2^–ΔΔCt^ method [50–53]. The sequences of the qPCR primers are listed in Supplemental Table 1.

### Construction of miR-223 and let-7a-1 Gaussia luciferase (GLuc) reporters

The GLuc reporters for miR-223 and let-7a-1 were constructed on the base of our recently characterized reporter vector pNRGLuc, which express Gaussia luciferase (GLuc) with a multiple cloning linker at the 3′-end of GLuc coding region [54, 55]. As human *ARRB1* and *LIN28B* are well established targets for miR-223 and let-7a-1, which contain miR-223 and let-7a-1-binding sites at their 3′-UTRs, respectively, we PCR amplified the 3′-untranslated sequences of human *ARRB1* (or BUTR) and *LIN28B*, and subcolned them into pNRGLuc vector, resulting in pNRGLuc-BUTR (or GLuc-BUTR) and pNRGLuc-LIN28B (or GLuc-LIN28B), respectively. For the control reporters, mutations were introduced to the miR-binding sites and yielded pNRGLuc-BUTR Mut (or GLuc-BUTR Mut) and pNRGLuc-LIN28B Mut (or GLuc-LIN28B Mut). All PCR amplified sequences were verified by DNA sequencing.

### Gaussia luciferase (GLuc) reporter assay

The GLuc reporter assays were carried out as described [56–58]. Briefly, exponentially growing HEK-293 and HCT116 cells were seeded in 12-well cell culture plates and coinfected with different combinations of miR expression vectors and/or the GLuc reporter plasmids. At 72 h after transfection, the cell culture media were subjected to GLuc activity assays using the BioLux GLuc Assay Kit (NEB). Each assay condition was done in triplicate.

### Cell proliferation WST-1 assay

The WST-1 cell proliferation assay was performed as described [59–62]. Experimentally, exponentially growing cells were seeded in 60 mm cell culture dishes and transfected with miR expression plasmids or the control vector. At 16 h after transfection, the transfected cells were replated into 96-well cell culture plates at 30% confluence. Unseeded wells were added with culture medium and utilized as background controls. At the indicated time points, the premixed WST-1 (Takara Bio USA) was added to each well and incubated at 37 °C for 2 h. The plates were subjected to a microtiter plate reader to obtain absorbance reading at 450 nm. Each assay condition was done in triplicate.

### Crystal violet staining assay

The crystal violet cell viability assay was carried out as described [41, 63–65]. Briefly, subconfluent cells seeded in 35 mm cell culture dishes and transfected with miR expression plasmids or vector control. At 3 days after transfection, the cells were subjected to crystal violet staining. Macrographic images of the stained dished were recorded. Each condition was done in triplicate.

### Cell cycle analysis

Cell cycle analysis was conducted as previously described [57, 66, 67]. Exponentially growing HEK-293 and HCT116 cells were seeded in 60 mm dishes and transfected with let-7a-1 expression plasmids or vector control. At 48 h post transfection, cells were collected and stained with the Magic Solution (containing 0.05% NP-40, 4% formaldehyde, 0.01 µg/ml Hoechst 33258 in PBS) for 30 min [68]. The stained cells were subjected to flow cytometry analysis using BD FACSCalibur-HTS. The flow cytometry data were quantitatively analyzed by FlowJo software. Each assay condition was performed in triplicate.

### Statistical analysis

The quantitative assays were performed in triplicate and/or repeated in three independent batches. Data were expressed as mean ± standard deviation. The one-way analysis of variance was used to analyze statistical significance. A value of *p* < 0.05 was considered statistically significant.

## Results

### The design of a novel mature miRNA expression system

Towards simplifying the exogenous expression of miRNAs, we designed two systems to express pMIRs and mMIRs, and then compared their functionality with the conventional miRNA expression system, in which the priMIR expression is accomplished by using a Pol II promoter (e.g., CMV) to drive a 200–500 bp genomic DNA fragment containing miRNA transcript unit (Fig. 1a, *a*). To express pMIRs, we constructed the pMIR expression system, in which human U6 promoter is used to drive the expression of pre-miR sequence followed by a string of five UUUUU (or TTTTT in the diagram) as the transcriptional termination signal (Fig. 1a, *b*). For the expression of mMIRs, we explored the possibility to use a converging U6/H1 dual promoter-driven system, which has been successfully used to express siRNAs [39, 40]. However, we had to overcome the asymmetric nature or imperfect complementarity of the 5p-miR and 3p-miR sequences of a given miRNA by inserting the transcription stop signals (a string of TTTTTAAAAA) between the 5p-miR (in sense direction) and 3p-miR (in antisense direction) sequences to terminate the transcription of 5p-miR and 3p-miR, respectively. Thus, in this converging dual-promoter system, the mMIR expression is achieved by using the convergent U6 and H1 promoters to drive the expression of mature miR-5p and miR-3p in opposing directions, respectively, with the respective transcriptional termination signal (TTTTT or UUUUU) separating the U6 and H1 expression units (Fig. 1a, *c*). Both pMIR and mMIR expression systems have been engineered in the adenoviral shuttle vector and retroviral transfer vector so they can easily be used to generate recombinant adenoviruses or retroviruses (Supplementary Figs. 1 and 2).

To evaluate the functionality of mMIR expression system in comparison to the conventional pri-miRNA and pMIR expression systems, we carried out a proof-of-concept study and focused on two well-studied miRNAs, hsa-miR-223 and hsa-let7a-1. Briefly, we constructed the pri-miR-223 expression vector by cloning the PCR amplified 200 bp human genomic DNA fragment containing miR-223 under the control of a CMV promoter, resulting in priMIR223. Similarly, the pMIR223 and mMIR223 were constructed to express pMIRs and mature miR-223, respectively (Fig. 1b, *a*). Using the same set of vectors, we constructed expression vectors for primary let-7a-1 (priMIRLET7A1), precursor let-7a-1 (pMIRLET7A1), and mature let-7a-1 (mMIRLET7A1), respectively (Fig. 1b, *b*).

### Exogenous expression of mature miR-223 can effectively inhibit target gene expression in human cells

**Fig. 2:**
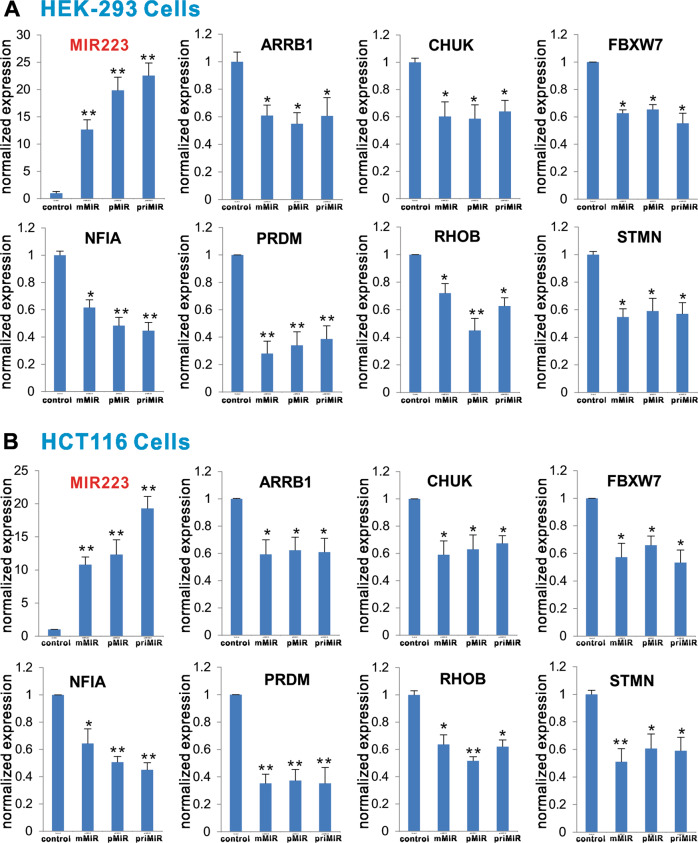
Comparison of target gene expression inhibition mediated by the three miR-223 expression systems. Confluent HEK-293 (**a**) and HCT116 (**b**) cells were transfected with empty vector (Control), mMIR223, pMIR223, or priMIR223. At 72 h after transfection, total RNA was isolated and subjected to TqPCR analysis. The expression of miR-223 mediated by the three expression systems was also assessed. *p < 0.05 and **p < 0.01 compared with the vector control group

It has been well established that miR-223 functions as an oncomiR and regulates numerous target genes in cell proliferation and other cellular functions [34, 69–71]. To test whether the mMIR223 expression vector would produce functional mature miR-223 and regulate some of the target gene expression in a similar fashion to the conventional expression system pMIR223, we transfected these three vectors into two human cell lines, HEK-293 and HCT116. Using qPCR analysis, we found that all three vectors expressed high levels of miR-223 in HEK-293 cells, compared with the vector control group, although the expression level in the priMIR223 group was seemingly higher than that of the pMIR223 and mMIR223 groups (Fig. 2a). We selected seven well-characterized miR-223 target genes, including *ARRB1, CHUK, FBXW7, NFIA, PRDM, RHOB*, and *STMN* [34, 70], and analyzed their expression levels in the transfected HEK-293 cells. Our results indicate that, compared with the vector control group, both pMIR223 and mMIR223 groups exhibited significantly reduced expression levels of the tested seven target genes (Fig. 2a). Moreover, the inhibition of the tested seven target genes mediated by both pMIR223 and mMIR223 vectors was as effective as that of the conventional primary miR-223 expression vector priMIR223 (Fig. 2a).

We conducted the same assays in HCT116 cells. Our results showed that high levels of exogenous miR-223 expression were readily detected in HCT116 cells, although the priMIR223 vector seemingly again mediated the highest level of miR-223 expression (Fig. 2b). While the exact cause(s) of the differences in expression levels remain to be understood, it may reflect the fact that CMV promoter may be much stronger than U6 and/or H1 promoters. Upon examining the expression levels of the selected seven target genes, we found that all three vector transfected groups effectively inhibited the expression of these target genes (Fig. 2b). Similar to the results obtained in HEK-293 cells, pMIR223- and mMIR223-mediated inhibition of the target gene expression was statistically insignificant with that of priMIR223. Collectively, these results suggest that our pMIR223 and mMIR223 expression systems may produce functional miR-223 as effectively as the commonly used primary miR223 system.

### Exogenous expression of mature miR-223 specifically inhibits the miR-223 reporter activity in human cells

**Fig. 3:**
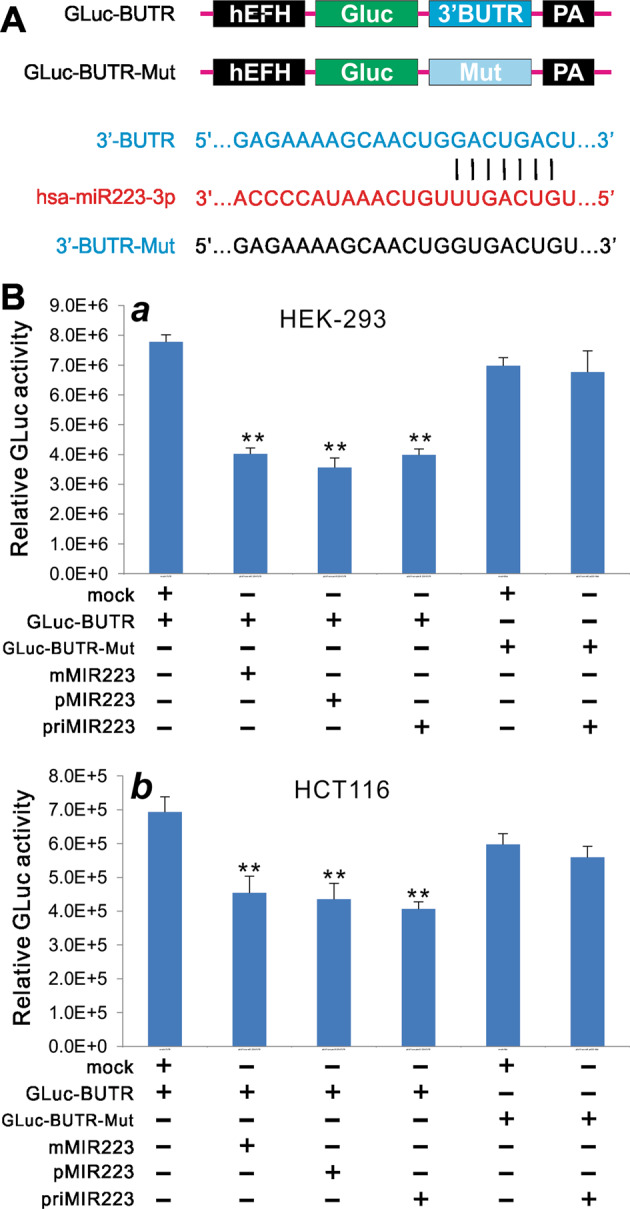
Comparison of the inhibition of miR-223 reporter activity mediated by the three miR-223 expression systems. **a** Construction of the miR-223 Gaussia luciferase (GLuc) reporter using the human β-arrestin 3′-UTR (3′BUTR) that contains a miR-223-binding site. A mutant-binding site reporter (3′BUTR-Mut) was also constructed as a control. **b** Subconfluent HEK-293 (*a*) and HCT116 (*b*) cells were cotransfected with different combinations of reporter vectors and miR-223 expression plasmids. GLuc activity was assessed at 72 h after transfection. Assays were done in triplicate. **p < 0.01 compared with the GLuc-BUTR + vector control (mock) group

To further test the functionality of mMIR223 vector, we constructed the GLuc-based miR-223 reporter, GLuc-BUTR, by cloning a 200 bp miR-223-binding site-containing fragment of the 3′-UTR of human *ARRB1* transcript (BUTR) into the linker sites of our previously reported miRNA reporter vector pNRGLuc [34]. We also constructed a control reporter by mutating the miR-223-binding site of BUTR (i.e., GLuc-BUTR-Mut) (Fig. 3a). When the GLuc-BUTR reporter or the mutant control reporter GLuc-BUTR Mut was cotransfected with the miR-223 expression vectors into HEK-293 cells, we found the all three miR-223 expression vector groups exhibited significantly inhibitory effect on GLuc activities, compared with the mock vector control group (Fig. 3b, *a*). Furthermore, the inhibitory effect was rather specific as these vectors did not significantly inhibit the GLuc activity of the control reporter, which has the miR-223-binding site mutated (Fig. 3b, *a*). Similar results were obtained in HCT116 cells. We found all three miR-223 expression vector groups exhibited significantly inhibitory effect on GLuc activities in the GLuc-BUTR group, but not in the GLuc-BUTR-Mut group, compared with the mock vector control group (Fig. 3b, *b*). These results further suggest that the mMIR223 expression system may produce miR-223 as effectively as the conventional primary miR223 and pMIR223 expression systems.

### Exogenous expression of mature miR-223 effectively promotes cell proliferation

**Fig. 4:**
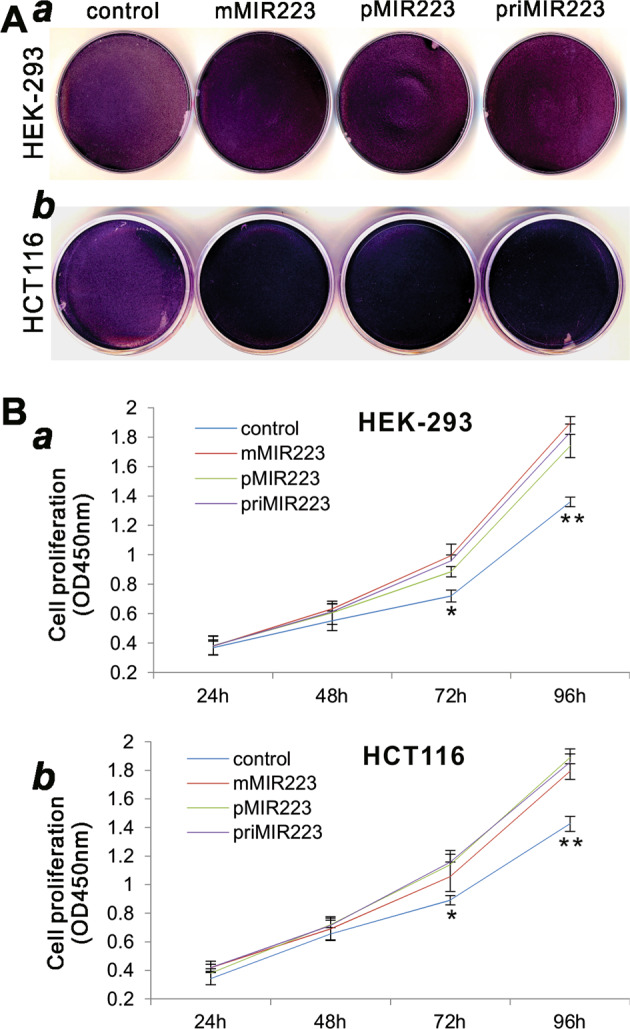
Comparison of the augmented cell proliferative activity mediated by the three miR-223 expression systems. **a** The crystal violet assay. Subconfluent HEK-293 (*a*) and HCT116 (*b*) cells were transfected with the indicated miR-223 expression plasmids or vector control. Cells were fixed for Crystal violet staining at 3 days after transfection. Assays were done in triplicate, and representative results are shown. **b** The WST-1 cell proliferation assay. Subconfluent HEK-293 (*a*) and HCT116 (*b*) cells were transfected with the indicated miR-223 expression plasmids or vector control. At the indicated time points, WST-1 substrate was added to each well and incubated for 2 h, followed by measuring the absorbance at 450 nm. Assays were done in triplicate. *p < 0.05 and **p < 0.01 compared with the vector control group

As miR-223 is considered as an oncomiR, we further analyzed the biological effects of the exogenously expressed miR-223 on cell viability and proliferation. When pMIR223 and mMIR223 vectors were transfected in HEK-293 and HCT116 cells, we found that, based on the crystal violet staining assay, cell densities increased significantly, compared with that of the vector control group in both cell lines (Fig. 4a, *a*, *b*). Consistent with the results obtained from the qPCR analysis of the target gene expression and the miR-223 reporter assay, the enhancement of cell density in pMIR223 and mMIR223 transfection groups was shown similar to that observed in the priMIR223 (Fig. 4a).

We also carried out the more quantitative WST-1 cell proliferation assay. When the three miR-223 expression vectors, pMIR223, mMIR223, and priMIR223, were transfected in HEK-293 and HCT116 cells, we found that cell proliferation rates were significantly increased at 72 and 96 h after transfection, compared with that of the vector control group in both cell lines (Fig. 4b, *a*, *b*). Thus, these results collectively demonstrate that the exogenous expression of miR-223 mediated by mMIR223 can achieve similar biological functions conferred by the commonly used priMIR priMIR223 or pMIR223 expression system.

### Exogenous expression of mature let-7a-1 (mMIRLET7A1) effectively inhibits target gene expression in human cells

**Fig. 5:**
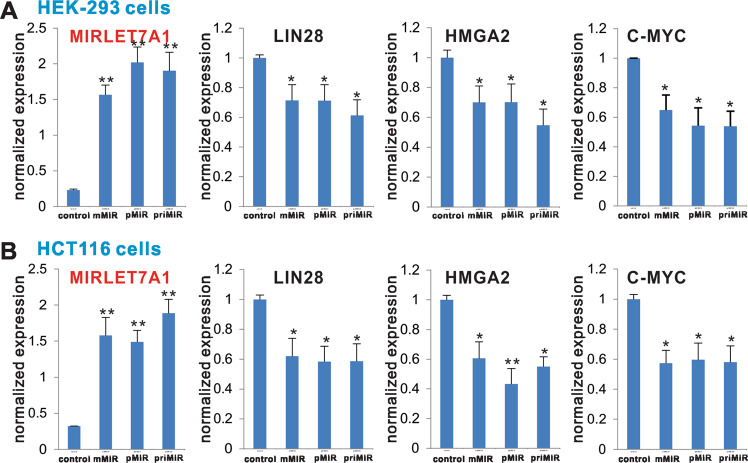
Comparison of target gene expression inhibition mediated by the three let-7a-1 expression systems. Confluent HEK-293 (**a**) and HCT116 (**b**) cells were transfected with empty vector (control), mMIRLET7A1, pMIR LET7A1, or priMIR LET7A1. At 72 h after transfection, total RNA was isolated and subjected to TqPCR analysis. The expression of let-7a-1 mediated by the three expression systems was also assessed. *p < 0.05 and **p < 0.01 compared with the vector control group

To ensure the general applicability of the reported pMIRs and mMIR expression vectors, we chose to test another well-studied miRNA let-7a-1 and constructed mature (mMIRLET7A1), as well as the conventional priMIR (priMIRLET7A1) and the precursor (pMIRLET7A1) expression vectors (Fig. 1b, *b*). We tested the three vectors in two human cell lines, HEK-293 and HCT116, respectively. Using qPCR analysis, we found that all three vectors expressed high levels of let-7a-1 in HEK-293 cells, compared with the vector control group (Fig. 5a). The expression of three let-7a-1 target genes, *LIN28, HMGA2*, and *C-MYC* [72–74], was effectively inhibited by mMIRLET7A1 at a comparable level with that of priMIRLET7A1 or pMIRLET7A1, compared with that of the vector control group (Fig. 5a).

The same assays were carried out in HCT116 cells. We found that high levels of exogenous let-7a-1 expression were readily detected in HCT116 cells (Fig. 5b). Upon examining the expression levels of the test three target genes, we found that all three vector transfected groups effectively inhibited the expression of these target genes (Fig. 5b). Furthermore, the inhibited expression of the target genes mediated by mMIRLET7A1 was statistically insignificant with that of priMIRLET7A1 or pMIRLET7A1. Thus, these results further suggest that the mMIRLET7A1 expression system may produce functional let-7a-1 as effectively as the conventional primary let-7a-1 or the pMIRLET7A1 expression system.

### Exogenous expression of mature let-7a-1 (mMIRLET7A1) specifically inhibits the let-7a-1 reporter activity in human cells

**Fig. 6:**
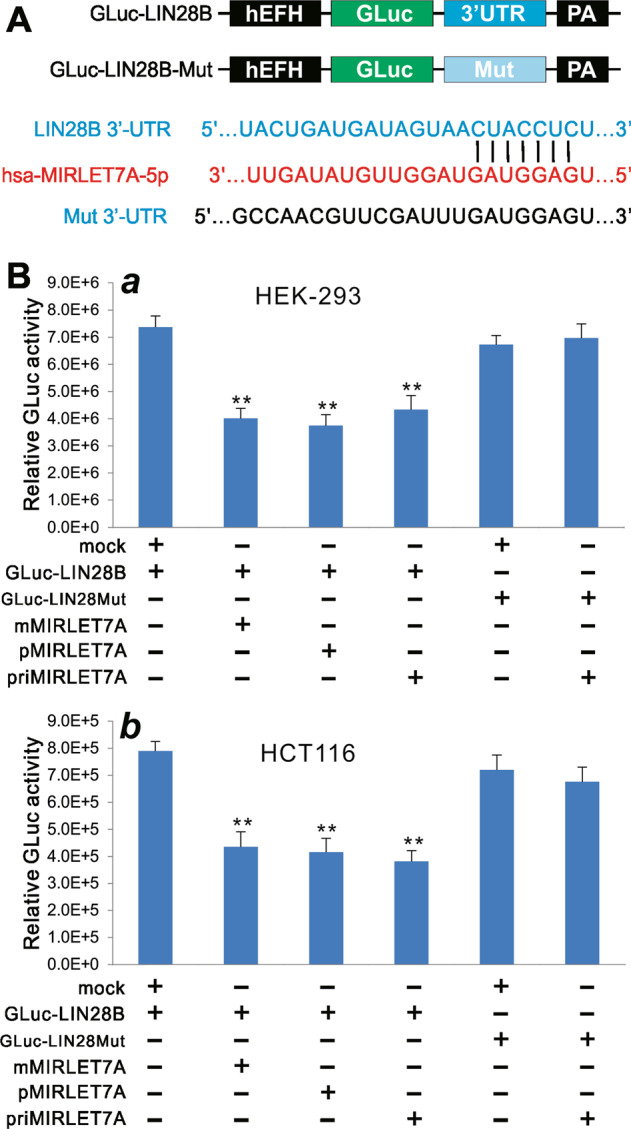
Comparison of the inhibition of let-7a-1 reporter activity mediated by the three let-7a-1 expression systems. **a** Construction of the let-7a-1 GLuc reporter using the human LIN28B 3′-UTR that contains a let-7a-1-binding site. A mutant-binding site reporter (Mut 3′-UTR) was also constructed as a control. **b** Subconfluent HEK-293 (*a*) and HCT116 (*b*) cells were cotransfected with different combinations of reporter vectors and let-7a-1 expression plasmids. GLuc activity was assessed at 72 h after transfection. Assays were done in triplicate. **p < 0.01 compared with the GLuc-LIN28B + vector control (mock) group

We further constructed a GLuc-based let-7a-1 reporter, GLuc-LIN28B, by cloning a 525 bp let-7a-1-binding site-containing fragment of the 3′-UTR of human *LIN28B* transcript into the linker sites of pNRGLuc [34]. A control reporter was also constructed by mutating the let-7a-1-binding site (i.e., GLuc-LIN28B-Mut) (Fig. 3a). When the GLuc-LIN28B reporter or the mutant control GLuc-LIN28B-Mut was cotransfected with the let-7a-1 expression vectors into HEK-293 cells, we found all three let-7a-1 expression vectors exhibited significantly inhibitory effect on GLuc activities, compared with the mock vector control group (Fig. 6b, *a*). Furthermore, the inhibitory effect was shown specific as these vectors did not significantly inhibit the GLuc activity of the mutant control reporter (Fig. 6b, *a*). Similarly, in the HCT116 cells we found the three let-7a-1 expression vectors exhibited significantly inhibitory effect on GLuc activities of the GLuc-LIN28B reporter, but not in the GLuc-LIN28B-Mut control reporter, compared with the mock vector control group (Fig. 6b, *b*). These findings further indicate that mMIRLET7A1 expression system can produce functional let-7a-1 as effectively as the conventional primary let-7a-1 or the pMIRLET7A1 expression system.

### Exogenous expression of mature let-7a-1 (mMIRLET7A1) effectively inhibits cell proliferation and cell cycle progression

**Fig. 7:**
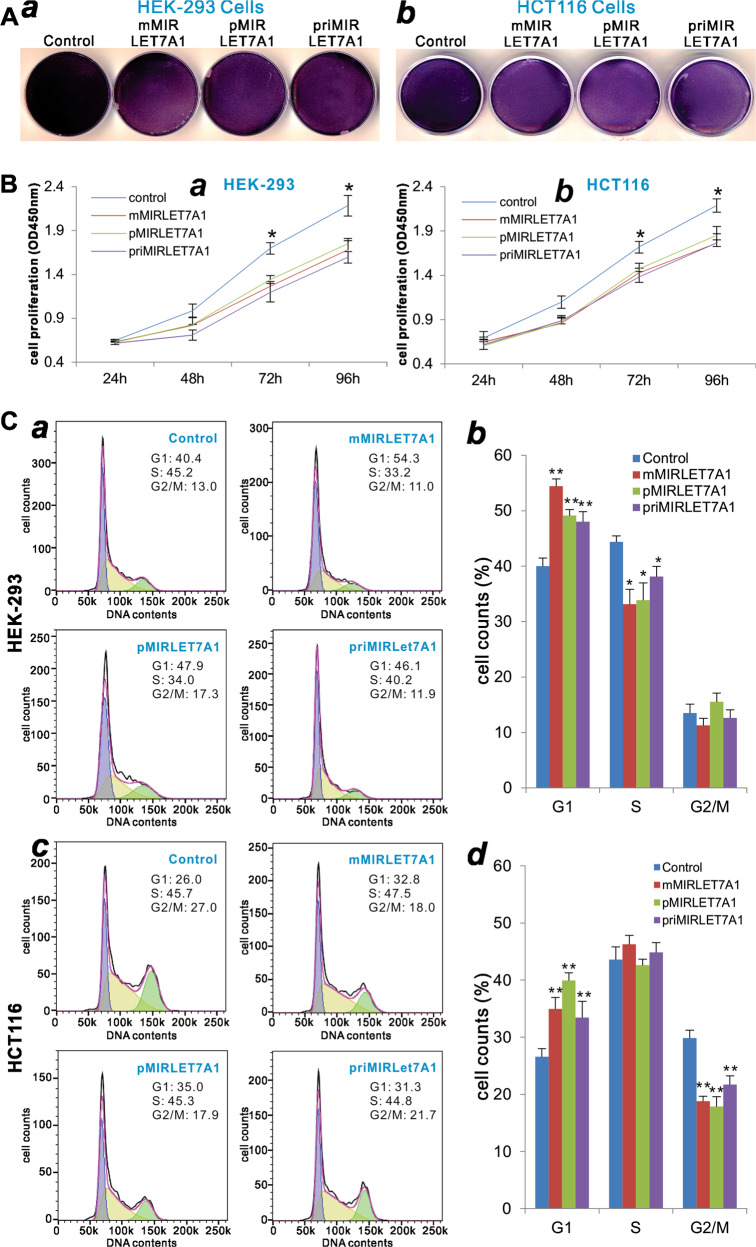
Comparison of the let-7a-1-inhibited cell proliferative activity mediated by the three let-7a-1 expression systems. **a** The crystal violet assay. Subconfluent HEK-293 (*a*) and HCT116 (*b*) cells were transfected with the indicated let-7a-1 expression plasmids or vector control. Cells were fixed for Crystal violet staining at 3 days after transfection. Assays were done in triplicate, and representative results are shown. **b** The WST-1 cell proliferation assay. Subconfluent HEK-293 (*a*) and HCT116 (*b*) cells were transfected with the indicated let-7a-1 expression plasmids or vector control. At the indicated time points, WST-1 substrate was added to each well and incubated for 2 h, followed by measuring the absorbance at 450 nm. Assays were done in triplicate. *p < 0.05, **p < 0.01 compared with the vector control group. **c** Cell cycle analysis. Subconfluent HEK-293 (*a*, *b*) and HCT116 (*c*, *d*) cells were transfected with the indicated let-7a-1 expression plasmids or vector control. At 48 h after transfection, cells were harvested and subjected to cell cycle analysis. Assays were done in triplicate, and representative results are shown (*a*, *c*). Quantitative analyses were carried out to determine the % cell counts in various phases (*b*, *d*). *p < 0.05 and **p < 0.01 compared with the vector control group

The let-7a-1 miRNA functions as a tumor-suppressor miRNA [72–75]. Here, we also analyzed the biological effects of the exogenously expressed let-7a-1 on cell viability, cell proliferation, and cell cycle progression. When mMIRLET7A1 vectors were transfected in HEK-293 and HCT116 cells, crystal violet staining assay indicates that the cell densities decreased significantly, similar to that of the priMIRLET7A1 or pMIRLET7A1 transfection group, compared with that of the vector control group in both cell lines (Fig. 7a, *a*, *b*).

The quantitative WST-1 cell proliferation assay also revealed that three let-7a-1 expression vectors, pMIRLET7A1, mMIRLET7A1, and priMIRLET7A1, were transfected in HEK-293 and HCT116 cells, we found that cell proliferation rates were significantly decreased at 72 and 96 h after transfection, compared with that of the vector control group in both cell lines (Fig. 7b, *a*, *b*). Collectively, these results demonstrate that the exogenous expression of let-7a-1 mediated by mMIRLET7A1 can achieve similar biological functions conferred by the commonly used priMIRLET7A1 or the pMIRLET7A1 expression system.

Lastly, we examined the effect of the exogenously expressed let-7a-1 on cell cycle progression. When transfected in HEK-293 cells, the mMIRLET7A1 vector, similar to the priMIRLET7A1 or pMIRLET7A1 vector, was shown to increase the % cells presented in the G1 phase, while decreasing % cells in the S phase, compared with the vector control (Fig. 7c, *a*, *b*). When transfected into the HCT116 cells, the mMIRLET7A1 vector, along with the priMIRLET7A1 or the pMIRLET7A1 vector, was shown to increase the % cells presented in the G1 phase (Fig. 7c, *c*, *d*). Interestingly, unlike in HEK-293 cells, the expression of exogenous let-7a-1 led to decreases of % cells in the G2/M phases, while no significant changes in the S phase (Fig. 7c, *ab* vs *cd*). Taken together, these results demonstrate that the mMIRLET7A1 vector can express biological active form of let-7a-1 miRNA, which is functionally indistinguishable with that expressed by the conventional primary let-7a-1 miRNA or the pMIRLET7A1 expression system.

## Discussion

In order to develop a user-friendly approach for the effective expression of functional miRNAs, we engineered a simplified strategy to express miRNAs by utilizing the converging U6/H1 dual promoters to drive the expression of mMIRs. Even though the converging U6/H1 dual promoter-driven system has been successfully used to express siRNAs [39, 40], the asymmetric nature or imperfect complementarity of the 5p-miR and 3p-miR sequences of a given miRNA poses a technical challenge. We overcame this challenge by inserting the transcription stop signals TTTTTAAAAA between the 5p-miR (in sense direction) and 3p-miR (in antisense direction) sequences to terminate the transcription of 5p-miR and 3p-miR, respectively.

By comparing with the commonly-used priMIR and pMIR expression systems, we demonstrated that the oncomiR hsa-miR-223 expressed in the mature (mMIR223) form effectively inhibited target gene expression, specifically suppressed miR-223 reporter activity, and promoted cell proliferation of HEK-293 and HCT116 cells, in a similar fashion comparable with that of the primary miR-223 or the precursor miR-223 (pMIR223) expression system. Similarly, the tumor-suppressor hsa-let-7a-1 expressed in the mature let-7a-1 forms effectively inhibited the expression of target genes, the reporter activity derived from LIN28B 3′-UTR, and cell proliferation and cell cycle progression, which were indistinguishable from the conventionally expressed primary let-7a-1 or precursor let-7a-1. It is noteworthy that our mMIR expression vectors were constructed on the bases of retroviral transfer and adenoviral shuttle vectors. Thus, the final miRNA expression constructs can be easily converted into recombinant retrovirus for stable expression or recombinant adenovirus for effective transient expression in vitro and in vivo. Taken together, our results demonstrate that the simplified mMIR expression system is user-friendly and reproducibly effective, which should be a valuable resource for studying miRNA functions and/or exploring potential applications of miRNA-based therapeutics.

It has long been a challenge to overexpress functional miRNAs in an effective and reliable fashion. In most cases, overexpression of miRNAs is accomplished by the use of chemically synthesized miRNA mimics, which are mostly made through commercial sources. While this approach is effective and has been widely used, the efficacy of miRNA mimics is transient in nature and relies on transfection efficiency, which may vary drastically in different cell lines. Furthermore, it may be technically challenging and/or preventively expensive to use synthesized miRNA mimics for in vivo animal studies.

Several strategies have been used to express miRNAs in the cells and potentially in animals. One commonly employed miRNA expression system is to use a Pol II promoter, such as CMV, SV40, or CMV enhancer/β-actin (CA) promoter, or Pol III promoters to drive the expression of a priMIR, which is contained in a 200–500 bp genomic DNA fragment [76, 77]. It has been attempted to express multiple miRNAs or shRNA-like stem-loop structures in a single transcript or polycistronic transcript [78–81]. The advantages of this system are the strong transcriptional activity of Pol II promoter and the easy incorporation of induciblity of miRNA expression. In a thorough examination of Pol II- and Pol III-driven expression of shRNA-like stem-loop pri-miRNAs, Furukawa et al. [77] found that, if the construct containing genomic sequences of 100 bp or less flanking the miRNA-encoded region was expressed, the CMV, CMVi, and CA promoters were more effective than the PGK promoter. However, it is not automatically guaranteed that the priMIRs is effectively processed. In fact, it was shown that miRNA precursors might fail to be efficiently processed to generate the mature forms of miRNAs in some types of cells or under certain conditions [77]. Nonetheless, the U6-driven stem-loop structure of miRNA produced a larger difference between the 5′- and 3′-strand of the miRNA duplex in miRNA-mediated suppressive effects on reporter gene expression than the pol II promoter-derived pri-miRNA [77], suggesting that U6 promoter may be more appropriate for driving the expression of priMIRs. Our results have demonstrated that the pMIR U6-driven pri-miRNA expression is highly effective and functional.

Another commonly used method takes advantage of the intronic miRNA expression systems [35–37]. This strategy was originated from the discovery of intronic miRNAs (also termed “mirtron”), which are derived from the processing of introns and differ from the intergenic miRNAs in the requirement of RNA polymerase II and splicing machinery components for their biogenesis [82–84]. Such short intronic hairpin miRNAs can bypass Drosha cleavage step, and are instead processed by the splicing machinery and lariat-debranching enzyme to yield pMIR-like hairpins [82–85]. Several variations of such intronic miRNA expression system have been devised over the years. Wu et al. [35, 36] constructed the miRNA and shRNA expression vectors, such as pSM155 and pSM30, by placing miRNA-based artificial miRNA expression cassettes inside of synthetic introns to take advantage of miRNA processing and RNA splicing mechanisms. More recently, a multiplexed miRNA and transgene expression platform exploiting native intronic miRNA loci was reported to accomplish simultaneous repression and expression of protein coding sequences, which may facilitate applications of simultaneous gene silencing and corrective transgene expression in some cases, such as polygenic therapeutic targets, drug resistance genes, viral and host genes involved in the viral life cycle, or oncogenes and tumor suppressor genes [37]. However, the identification of natural human intronic miRNA sequences may be challenging, if not impractical.

A major challenge for exogenously overexpressing priMIR, shRNA-like, or pMIRs is that the production efficiency of mMIRs is dependent on the endogenous miRNA processing and can cause cytotoxicity due to oversaturation of the RNAi processing machinery [37, 38, 86]. In fact, Ago2 was identified as the primary rate-limiting determinant of both in vitro and in vivo RNAi efficacy, toxicity, and persistence [86]. It was shown that in a mouse study, vector-based Ago-2/Xpo-5 co-expression enhanced U6-driven shRNA silencing of exogenous and endogenous hepatic targets, reduced hepatotoxicity, and extended RNAi stability by more than 3 months [86]. Ago2 overexpression was shown to significantly boost mRNA silencing efficiency in cell culture by up to tenfold, accompanied with reduced off-targeting effects [87].

Taking together, here we developed a novel and simplified strategy to effectively express mMIRs. This system should theoretically bypass most of the siRNA/miRNA processing machinery, including the involvement of Drosha, Expotin-5, and Dicer1, although it still requires the Ago proteins to fulfill gene silencing function. Therefore, our mMIR expression system is highly customizable and should broadly improve the in vitro and in vivo applications of miRNA experimentations.

## Supplementary information

Suppl. Table 1

Suppl. Figure 1

Suppl. Figure 2
