# Supplementary material for: A simplified system for the effective expression and delivery of functional mature microRNAs in mammalian cells
Source: Cancer Gene Ther. 2019 Jun 20;27(6):424–37. doi: 10.1038/s41417-019-0113-y (PMC6923634; doi:10.1038/s41417-019-0113-y)
Supplement: Supplementary file 1 — Suppl. Table 1 [file 41417_2019_113_MOESM1_ESM.pdf]

Supplemental Table 1. List of miRs Primers and Their Use

Gene	Primer Sequences	Use
hsa-let-7a-1	TCGATGAGGTAGTAGGTTGTATAGTTTTTTTAAAAAGAAAGACAGTAGATTGTATAG<br>AGCTCTATACAATCTACTGTCTTTCTTTTTAAAAAAACTATACAACCTACTACCTCA	miRNA expression vector cloning
hsa-plet-7a-1	CGCGTGAGGTAGTAGGTTGTATAGTTTTAGGGTCACACCCACCACTGGGAGATAACTATACAATCTACTGTCTTTCTTTT<br>AGCTAAAAAGAAAGACAGTAGATTGTATAGTTATCTCCCAGTGGTGGGTGTGACCCTAAAACTATACAACCTACTACCTCA	
hsa-prilet-7a-1	GGAGGATCCGGAGCGGATTCAGATAACCAAGC<br>GTCGTCGACGACAAGAAGCAAAAGGTTTCCCC	
hsa-miR-223	TCGACGTGTATTTGACAAGCTGAGTTTTTTTAAAAATGGGGTATTTGACAAACTGACA<br>AGCTTGTCAGTTTGTCAAATACCCCATTTTTAAAAAAACTCAGCTTGTCAAATACACG	
hsa-pmiR-223	CGCGCGTGTATTTGACAAGCTGAGTTGGACACTCCATGTGGTAGAGTGTCAGTTTGTCAAATACCCCATTTTT<br>AGCTAAAAATGGGGTATTTGACAAACTGACACTCTACCACATGGAGTGTCCAACTCAGCTTGTCAAATACACG	
hsa-primiR-223	TAAAAGCTTCCTCTAGGGTCACATCTCC<br>TAAACGCGTCTGGCAGTCCATTCGTCA	
ARRB1 3'UTR	TAGACCCCAGGAGTAGAGAAAAGCAACTGGTGACTGTTTCTTACCAGCAGTTACCT<br>TACAAGGTAGCTAAGATGTCACAGCCTGACAGAACTGAAATATACTTGGGTGAGTC	miR reporter cloning
LIN28B 3'UTR	GAAGAATTCGCTCAGGGAACATACCATGTAATA<br>ACGACGCGTTGTTTGGAGACTTTGCTAACTAAAACG	
hsa let-7a-1-5p	GTCGTATCCAGTGCAGGGTCCGAGGTATTCGCACTGGATACGACAACTAT	RT reactions
hsa miR-223-3p	GTCGTATCCAGTGCAGGGTCCGAGGTATTCGCACTGGATACGACTGGGGT	
human HMGA2	CCTCGCTTCCCTCCTCCT<br>GCCACCATCAACACCGGA	qPCR
human LIN28B	GGTAGCAGGGAGAGGGGT<br>GCTTCAGGGCAGCACAGA	
human c-MYC	CCACCTCCAGCTTGTACCTG<br>GAGCAGAGAATCCGAGGACG	
human NFIA	ATTCTCCGCTCTGTCTCACC<br>TCATCCTTCACGGCTCTCTC	
human CHUK	AGCGAGCAGATGACGTATGG<br>CTGACGTCTTCCATAGGGGC	
human PRDM1	CAACGCTCACTACCCCAAGT<br>TTGCTGTAGACAGGGCACAG	
human FBXW7	TGTGTGGCGGATCAGAGC<br>GGGGAAGGGCAGGGAGTA	
human RHOB	GACCCGTGTCCCACAAGG<br>CAGCTGGACGCACACAGA	
human STMN1	AAAGAGAACCGAGAGGCACAA<br>GGTAATCAATGCAGATTGGAGGC	

human ARBB1	GACAAAGGGACCCGAGTGTT<br>GCAGGTCAGCGTCACATAGA	
hsa-miR-223-3p	AGCCGTGTCAGTTTGTCAAAT<br>GTGCAGGGTCCGAGGTATTC	
hsa-let-7a-1-5p	GCGGCTGAGGTAGTAGGTTGT<br>GTGCAGGGTCCGAGGTATTC	
